# Evolving Clinical Applications of Tissue Transcriptomics in Kidney Disease

**DOI:** 10.3389/fped.2019.00306

**Published:** 2019-07-23

**Authors:** Andrea L. Oliverio, Tiffany Bellomo, Laura H. Mariani

**Affiliations:** ^1^Division of Nephrology, Department of Internal Medicine, University of Michigan, Ann Arbor, MI, United States; ^2^University of Michigan Medical School, Ann Arbor, MI, United States

**Keywords:** transcriptomics, single cell transcriptomics, nephrotic syndrome, glomerular disease, chronic kidney disease

## Abstract

Nephrotic syndrome is classically categorized by the histopathology with examples including focal segmental glomerulosclerosis (FSGS) and minimal change disease. Pediatric patients are also classified by whether their nephrotic syndrome is sensitive to, dependent on, or resistant to steroids. However, this traditional classification system overlooks the frequent clinical conundrum when, for example, one patient with FSGS responds briskly to steroids, and another quickly progresses to end stage kidney disease despite therapy. Two patients may have similar histopathologic appearances on kidney biopsy but entirely different clinical characteristics, rates of progression, and treatment responses. Transcriptional regulation of gene activation and posttranscriptional processing of mRNA may drive the unique and heterogeneous phenotypes which are incompletely understood in kidney disease and are a recent focus of research. Gene expression profiles provide insight on active transcriptional programs in tissues, are being used to understand biologic mechanisms of progressive chronic kidney disease, and may help to identify patients with shared mechanisms of kidney damage. This mini-review discusses clinically relevant techniques of bulk tissue and single cell transcriptomics, as well as strengths and limitations of each methodology. Further, we summarize recent examples in kidney research achieved through transcriptomics. This review offers an outlook on the role of transcriptomics in an integrative systems biology model with the goal of defining unique disease subgroups, finding targets for drug development, and aligning the right drug with the right patient.

## Introduction

Nephrotic syndromes are kidney diseases which result in proteinuria, hypoalbuminemia, edema, and hyperlipidemia. Traditionally nephrotic syndrome is categorized into diagnoses by their histopathology (minimal change disease, focal segmental glomerulosclerosis (FSGS), membranous nephropathy) or their response to steroids, however pathology and clinical phenotype do not predict or uniquely correlate with rate of progression or response to therapy. The transcriptome refers to all of the RNA expressed from genes of a particular organism, tissue, or cell type. Understanding the transcriptome can help elucidate the crucial link between genotype and phenotype, as gene expression and control of gene expression can vary under different states of health and disease. In addition, progressive kidney diseases may upregulate particular common transcriptional pathways—allowing scientists to identify prognostic biomarkers and develop interventions targeted at these shared pathways in chronic kidney disease (CKD). By defining kidney diseases more granularly than their histopathology and clinical presentation and incorporating data from transcriptomics as well as genomics, proteomics, and metabolomics—scientists work toward advancing the field of nephrology toward targeted treatments for kidney disease.

In this mini-review, we briefly review techniques used to analyze the transcriptome, specifically comparing and contrasting techniques that use “bulk” methods profiling the mRNA expression from whole tissue (in our case kidney biopsy tissue) vs. “single cell” methods profiling the mRNA expression within individual cells extracted from tissue or biofluids, with the goal of describing how these techniques are being used to improve our understanding and treatment of kidney disease.

## Bulk Transcriptomic Profiling

### Brief Methods and Techniques

DNA and RNA microarray technology emerged in the mid-1990s (1, 2), facilitating large scale analyses of gene expression data. Microarray formation begins with a pre-specified set of nucleic acid probes which are designed to recognize a specific RNA sequence and are bound to glass slides. Target sequences from the patient samples are hybridized to the probes and are subsequently fluorescently labeled. Images are then analyzed and quantified for signal intensity—with increasing intensity proportional to the greater abundance of the transcripts and hybridization to the probes. These hybridization techniques are relatively inexpensive but the probes rely on an *a priori* understanding of the model organism's genome and transcriptome and must be regularly updated with known sequence data. When using microarray technology, whole tissues can be analyzed en masse or can be divided into compartments. In human kidney research, the glomerular compartment and the tubulointerstitial compartment are commonly separated through microdissection, and are then analyzed separately (3, 4).

Massive parallel sequencing, also called Next Generation Sequencing or deep sequencing, was subsequently developed in the early 2000s (5). This technique is now frequently used to study the transcriptome and referred to as RNA-sequencing (RNA-Seq) technology. RNA-Seq continues high output transcriptional analyses, and unlike microarray technology, it is unencumbered by the need for a prior understanding of the genomic sequence, but rather allows sequencing of all RNA in the sample. This allows for greater discovery of the transcriptome across the full genome, including identification of splice isoforms by directly sequencing the transcripts. An additional benefit to RNA-Seq technology is the greater detection range compared to microarrays: it can detect low levels of expression or very high levels with greater precision, unlike the microarray probes which become saturated. Different platforms can be used for sequencing, all of which sequence millions of short genome fragments in parallel. Ultimately, transcript reads are aligned back to a known genome in order to determine their differential expression (i.e., up- or down-regulation of transcripts) in tissues, or they can be used for transcript discovery and mapping when a reference genome does not exist (6).

### Examples of Bulk Transcriptomics Analysis in Clinical Disease

#### A Novel Non-invasive Biomarker for Disease Progression: Urinary EGF

CKD is estimated to affect 11–13% of the overall population worldwide (7). While pediatric patients are a small proportion of the CKD and end stage kidney disease (ESKD) population, they suffer from a higher burden of hospitalizations, cardiovascular disease (8) and death (9) when compared to healthy children. Kidney Disease: Improving Global Outcomes (KDIGO) guidelines categorize stages of kidney function by a calculation of estimated glomerular filtration rate (eGFR) and an assessment of microalbuminuria/proteinuria (10). Estimated GFR is derived from serum creatinine, and in pediatrics accounts for height (11). The categories delineated by KDIGO, CKD 1–5, are associated with specific risks for progression to ESKD, and patients with CKD 1 and 2 have the lowest risk for progression. However, serum creatinine is notoriously a late marker for loss of renal function (12), and traditional formulas to estimate GFR perform poorly at the earlier stages of CKD (13). Thus, the challenge is to find patients who are at high risk to progress in early stages of their disease, so that interventions can be most efficacious. While histopathologic features on kidney biopsy can help predict outcomes independent of serum creatinine and estimated GFR (14, 15), the invasive and high risk nature of a kidney biopsy and high prevalence of CKD make this an untenable strategy for early detection. Developing early, non-invasive biomarkers is imperative to early detection and treatment.

Using microarray technology on kidney biopsy tissue, Ju et al. identified urinary epidermal growth factor (EGF) as an independent predictor of CKD progression (16). EGF is largely expressed in the thick ascending limb of Henle and distal tubule cells of the kidney (17), has pro-proliferative and anti-apoptotic functions, and enhances regeneration of renal tubule cells and recovery from injury (18). Ju's study used kidney biopsy tissue from a European biobank (European Renal cDNA Bank: ERCB) to identify intra-renal transcripts associated with eGFR at the time of biopsy. This work found the EGF transcript to be amongst those with greatest predictive performance for patient eGFR at time of biopsy. EGF expression was also shown to be highly specific to kidney tissue when compared to non-kidney tissue expression data and has known biology relevant to CKD progression. Intra-renal mRNA expression and correlation with eGFR was validated in additional ERCB patients as well as in a North American cohort (Clinical Phenotyping Resource and Biobank Core). Following this validation in kidney tissue, *urinary EGF protein* was measured and normalized to urinary creatinine, similar to the urine protein to creatinine ratio used in clinical practice today. The uEGF/Cr ratio was interrogated as a potential non-invasive marker for tissue-based RNA expression in three independent cohorts. uEGF/Cr was found to significantly correlate with intrarenal EGF transcript expression and eGFR at time of biopsy. uEGF/Cr importantly was also associated with eGFR slope and added predictive power to standard models to predict progression to ESKD. This relationship has been validated in several other studies. Betz et al subsequently showed that a lower uEGF/Cr was associated with rapid decline in renal function in a cohort of patients with diabetes with normoalbuminuria and preserved eGFR (19). In a cohort of 117 children with confirmed Alport syndrome, Li et al demonstrated that children with Alport syndrome showed significantly lower uEGF/Cr when compared to 146 age-matched healthy children (20). Again in this population, uEGF/Cr correlated tightly with eGFR (*r* = 0.75, *p* < 0.001), eGFR slope, and “progressors”—those who advanced to a higher CKD stage within the study follow up—were more likely to have low uEGF/Cr. Most recently, in a cohort of 623 children, low uEGF/Cr was associated with increased risk of CKD progression (21).

In summary, low uEGF/Cr level is a robust predictor of eGFR in cross-section, and it has also been shown to be a promising early biomarker to predict progression of kidney disease across several cohorts and disease types, including children. In many kidney diseases, EGF may be a general marker for tubular health, not captured by serum creatinine and urine protein. In this example, transcriptomic data was used to identify a novel biologic process and then applied to develop a non-invasive marker which reliably captures intra-renal transcriptional activity, and predicts kidney disease progression.

#### A Novel Mechanism and Treatment Target: JAK-STAT in Diabetic Nephropathy

Transcriptomics has also found a role in elucidating mechanisms of disease which escape traditional animal models. While mouse models of diabetic nephropathy mimic early stage disease in humans, they fail to replicate the progressive histopathological, and biochemical changes that are classic for advanced diabetic nephopathy (22). Using microarray technology, Berthier et al. compared transcriptional patterns from kidney tissue of early diabetic disease, progressive kidney disease, and controls (kidney donor tissue and patients with minimal change disease), finding that Janus kinases-signal transducer and activator of transcription (JAK-STAT) signaling pathways are differentially expressed in different kidney tissue compartments (glomerular vs. tubulointerstitial), and at different stages of diabetic kidney disease (early vs. late and progressive) (23). In particular, Jak-2, a protein that is part of the JAK-STAT signaling pathway, expression was upregulated in the glomerular compartment of humans with early diabetic nephropathy, and in late diabetic nephropathy Jak-2 expression is downregulated in the glomerular department and upregulated in the tubulointerstitial compartment. Tubulointerstitial Jak-2 expression was strongly, inversely correlated with eGFR of patients with diabetic nephropathy. However, Jak-2 mRNA and protein expression was not enhanced in two mouse models of diabetes when compared to control mice, suggesting that differences in JAK-STAT pathway signaling between humans and mice may be one reason why mouse models fail to replicate advanced diabetic nephropathy (23). JAK inhibitors have been developed for other diseases, and this work, along with that of others (24, 25), laid the foundation for trials of JAK inhibition for diabetic nephropathy. A recent phase II trial of baricitinib, an oral inhibitor of JAK1 and JAK2, was found to decrease albuminuria at 24 weeks when compared to placebo in patients with diabetic kidney disease (26). Applying the knowledge gained from studies in diabetic nephropathy, and specifically relevant to pediatrics and nephrotic syndrome, components of the JAK-STAT signaling pathway have also been shown to be upregulated in humans with biopsy-proven FSGS, and that the changes in JAK-STAT are associated with key clinical features among patients with FSGS, suggesting a role in pathogenesis as well as a potential target for treatment (27).

In this example, transcriptomic data from kidney biopsy was used to identify a targetable disease pathway in diabetic nephropathy that led to a successful early stage clinical trial. Insights gained from this foundational work have been applied to other kidney diseases such as FSGS, as well as models of polycystic kidney disease (28) and unilateral ureteral obstruction models of renal fibrosis (29), potentially providing a treatment target in a variety of kidney diseases.

### Strengths and Limitations of Bulk Tissue Transcriptomics

Bulk tissue transcriptomics has been used to identify biomarkers, novel mechanisms of disease, and potential targets for treatment. However, using tissue in bulk, such as tissue from a renal biopsy, to evaluate gene expression poses challenges because of the admixture of many cell types. While bulk methods advance our understanding of global transcriptional changes in the whole tissue, elucidating transcriptional changes in particular cell types remains a challenge. Bioinformatic methods have been developed to infer cell composition from bulk expression data based on unique cell markers, but characterizing single cell expression relies on inference based on marker gene expression levels, rather than direct measurement (30). Methods of amplifying transcripts from specific cell types are subject to biases from the amplification technique. Single cell transcriptomic techniques, discussed in detail in the second half of this review, are a recent advancement designed to address this issue.

## Single Cell Transcriptomic Profiling

While bulk sequencing is useful for identifying gene expression patterns across a whole tissue or tissue compartment, single cell sequencing techniques allow researchers to identify gene expression patterns of a single cell type within a whole tissue.

### Brief Methods and Techniques

Microdissection and sorting by flow cytometry to process bulk tissue into single cells for RNA analysis are limited by the inability to reliably purify a single cell type and interrogate a single cell's transcriptome. In other words, the resulting transcriptomic data is an average of RNA transcripts from a mixed group of cells (31). Newer sequencing methods rely on dissociating the tissue and encapsulating single cells in individual spaces (aka “droplets”), where each space contains everything necessary to label and then subsequently sequence the individual RNA transcripts of that single cell (32). These are referred to as “droplet-based” techniques. Droplet-based sequencing technology allows researchers to utilize that expression data from individual cells to cluster related cell types from a heterogeneous tissue, like the kidney. In this way, researchers can identify rare cell populations, discover new cell types, and track dynamic cellular processes (31, 33). Researchers can follow major biological mechanisms to identify cell-specific gene expression that may mediate tissue development, differentiation, and disease progression, which is more challenging with bulk sequencing techniques (31, 34).

**Figure 1:**
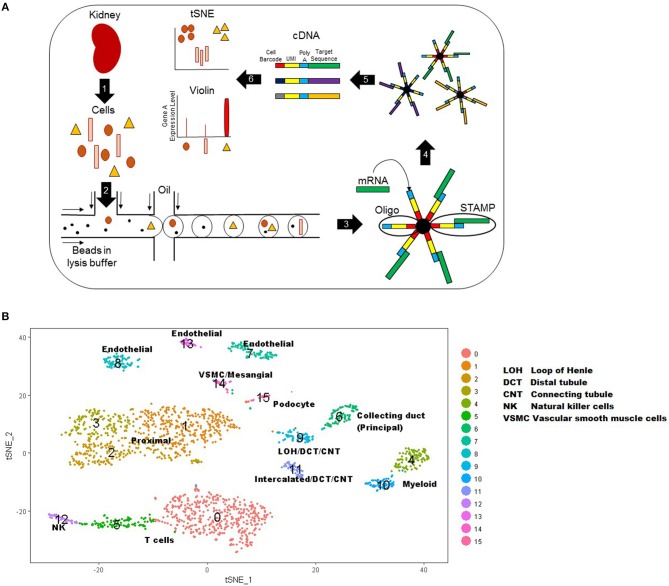
**(A)** Schematic of DropSeq workflow stepwise. Step 1: kidney biopsy tissue is digested into live single cells. Step 2 cells: cells, lysis buffer, and beads are injected into the microfluidic device where oil flowing across the stream pinches off droplets. Step 3: the lysed cell releases RNA for capture by bead primers. Step 4: droplets are broken to release beads with their cell-specific RNAs. Step 5: mRNA is reverse transcribed and cDNA is amplified for sequencing. Step 6: Data that has been mapped to the genome is analyzed to create a tSNE or Violin plot. UMI: Unique molecular identifier. STAMPs: Single Cell Transcriptomes attached to Microparticles. **(B)** T-Distributed Stochastic Neighbor Embedding (tSNE) plot representation of cell types in kidney tissue. Each cell is represented by a single point and points are grouped by similar gene expression profiles. In this plot, 15 clusters were identified and assigned a color as shown on the right. These 15 clusters were labeled as a specific cell type based on known genetic expression markers.

Single cell RNA sequencing involves five basic steps: single-cell isolation, cell lysis, reverse transcription of mRNA, amplification, and library generation (31, 35–37). In this review, we will specifically focus on the single cell droplet sequencing method named DropSeq. Figure 1A displays the steps of this technique. There are several mechanical and enzymatic methods used to dissociate live, single cells from tissue (31, 37). After single cell dissociation, a microfluidic device uses laminar flow to combine the suspension of single cells with a suspension of oligonucleotides (short sequences which can bind the mRNAs from that cell) attached to microparticles (beads) into single nanoliter droplets. Each bead has a unique identifier sequence to identify a single cell, and each oligonucleotide within a bead has a Unique Molecular Identifier (UMI) that identifies the single mRNA after amplification. After the droplets with one bead and one cell are created, the cells are lysed to release mRNA that is immediately captured by oligonucleotide primers. The new hybridized sequences are called Single Cell Transcriptomes attached to Microparticles (STAMPs). STAMPs are reverse transcribed, amplified, and sequenced in one reaction. Data analysis allows each mRNA transcript to be assigned by their cell barcode to a single cell type.

### Displaying Data From Single Cell Techniques

One of the most common graphical representations of single cell data is a T-Distributed Stochastic Neighbor Embedding (tSNE) plot, which is useful for visualizing cell populations (38). In a tSNE plot, cells are represented by dots and are grouped by similar gene expression profiles (Figure 1B). The data is represented as isolated clusters and the grouped cell types (e.g., podocytes vs. proximal tubular cells) can be identified based on known gene expression markers. Another plot commonly used to display single cell data is a violin plot. This plot displays the expression level of a given gene (y axis) by cell type clusters (cluster group along the x-axis).

### Strengths and Limitations of Single Cell Techniques

The advantages of single cell techniques are the ability to identify rare and new cell types and trace developmental relationships between heterogeneous cells (e.g., which cells produce similar proteins, transporters or hormone receptors) and cellular states (e.g., how cells change with health and disease) (34). Methodologically, DropSeq is a high-resolution technique that reduces technical variability and reduces amplification bias (32, 36, 39). Other advantages include relatively inexpensive set-up, which is cost-efficient for large numbers of heterogeneous cell samples (36, 37). One potential disadvantage of DropSeq is an event called a doublet; two cells are combined in one droplet, thereby all subsequent RNA transcripts are identified as originating from a single cell, rather than two cells. Additionally, capture efficiency is on average only 5% of input cells: data loss occurs when a cell is injected into a droplet without a bead. Another disadvantage of DropSeq technique includes potential apoptosis or membrane damage from the single cell dissociation, which may alter the transcriptional profile of studied cells or release mitochondrial RNA transcripts falsely elevating total transcript expression (40). These undesired events can obscure the mapping of raw reads to the genome. Techniques have been developed to overcome harsh tissue digestion methods (41, 42) and false elevations in transcript numbers (40), and there are analytical ways to clean raw data, removing mitochondrial mRNA. Although DropSeq can identify a cell type through differential mRNA expression of a single cell, there is no way to directly identify cell types based on shape, size, lineage, or location within the tissue (32).

### Evolving Technology

One of the largest quality improvements made on DropSeq is a technique termed 10X genomics. 10x genomics is a completely automated technique that allows for higher sequencing depth and speed as well as increased yield, which is suitable for more sensitive, smaller samples (43, 44). However, this technology comes at a cost (34): 10X genomics is 3 times more expensive than DropSeq analysis (45). C1 Fluidigm is single cell RNA sequencing using a chip instead of a droplet design to capture cells for higher throughput (31, 36). Another variation (Cell Expression by Linear Amplification and Sequencing: CEL-seq) creates a more efficient process by using *in vitro* transcription to combine barcoding and amplication steps into a single reaction (46–49). Table 1 describes the strengths, limitations, and recent applications of these technologies as well as bulk techniques.

**Table 1:** Strengths, weaknesses, and recent applications of bulk and single transcriptomic techniques in kidney disease.

	**Strengths**	**Weaknesses**	**Applications**
**BULK TECHNIQUES**			
Microarray	Inexpensive High output	Rely on a prior understanding of the genome or transcriptome Reference library must be regularly updated	Urinary EGF as non-invasive biomarker (16) Differential expression of JAK-STAT pathway in diabetic nephropathy and FSGS, a target for treatment (23–27)
Massive parallel sequencing	Can detect very high and very low levels of transcripts Does not require prior understanding of genomic sequence Allows sequencing of all RNA in the sample	Admixture of cell types: Methods of amplifying transcripts from specific cell types are subject to biases from amplification	TGF-B1/Smad signaling pathway in renal fibrosis and inflammation (50, 51)
**SINGLE CELL TECHNIQUES**			
DropSeq	Relatively inexpensive and appropriate for heterogeneous samples	Low sensitivity Low capture efficiency requires a large amount of cells	Inhibition of non-muscle myosin II increases cyst formation in polycystic kidney disease (52)
Smart-seq2	High sensitivity Full length RNA sequencing provides high read coverage appropriate for alternative splice form detection	Expensive Increased time to sequence	Profiling collecting duct cells (53)
10x Genomics	Completely automated High sequencing depth High sensitivity	Expensive	Reducing expression of Dab2 in renal tubule cells protect mice from CKD (54) Validating kidney micro-organoid models (55)
C1 Fluidigm	Completely automated Chip design allows for higher throughput	Expensive Cell capture chamber is a fixed size and cannot accommodate samples with varying cell sizes	IFN gene signature in tubular cells correlated with clinical outcomes in lupus nephritis (56) Multi-drug therapy design in metastatic renal cell carcinoma (57)
CEL-Seq2	Increased sensitivity to DropSeq Increased efficiency: optimizes primers, reagents, clean-up, and library preparation	Biased to 3' end of genes	

### Examples of Single Cell Transcriptomics Analysis in Clinical Disease

#### Developing a Kidney Cell Atlas to Discover New Kidney Cell Types

Without an accessible all-encompassing classification system for kidney cell types, researchers have been relying on fragmented systems based on one aspect of cell identity: location, function, or genetic profile. To combine these classification systems to create an all-inclusive cell atlas, Park et al used droplet-based single-cell RNA sequencing to catalog mouse kidney cell types from over 57,000 cells from seven healthy mice (58). Analysis revealed 16 distinct cell clusters, and each cluster's identity was assigned by performing differential gene expression analysis and comparing those unique genes to known cell type specific markers. They also identified novel markers for the cell types. To confirm that human mendelian kidney diseases show cell type specificity translatable to murine cell types, a literature search was conducted for mouse homolog genes to human genes associated with monogenic inheritance of a specific phenotype (e.g., nephrotic syndrome or renal tubular acidosis). The mouse homolog genes identified were present in the same cell-specific clusters as human genes. For example, mouse homolog genes to human genes causing podocyte dysfunction and nephrotic syndrome were identified in the murine podocyte cluster and, similarly, mouse homolog genes to human genes causing intercalated cell dysfunction, and renal tubular acidosis were identified in the murine intercalated cell cluster. The murine kidney cell atlas will be integral in understanding of novel kidney cell types and disease pathogenesis. For example, three murine cell clusters did not express genes of a known cell type, prompting further investigation to classify these new cell types. In one cluster, genes known to be expressed in both intercalated cells and principle cells were present, suggesting a new transitional cell type. To confirm that these transitional cells originated from intercalated cells, Notch cell signaling was induced in intercalated cells, resulting in production of cells expressing both transitional and principle cell markers. To understand the functional consequence of this new transitional cell type in CKD, bicarbonate levels and transitional cell expression were measured in mouse model of CKD. Increased transitional cells were associated with worsening metabolic acidosis in the murine kidney. These data showed that intercalated to principle cell transition was dependent on Notch signaling and may be in the causal pathway of metabolic acidosis in CKD, which is an area for further study.

In this example, single cell data was used to comprehensively identify cell types within mouse kidney tissue. It allowed direct comparison of mouse and human kidney cell types to assess similarities and differences, critical for using mouse models to study human disease. It also identified novel cell types with potential implications for identifying biological processes, previously not appreciated in humans. The human cell atlas (www.humancellatlas.org) project is working to create reference maps of all human cells.

#### Understanding Novel Disease Transcriptional Signatures

Single cell transcriptomic techniques have been combined with another recent technologic advance: the ability to derive kidney organoids, a simplified cohort of human kidney cell types induced from pluripotent stem cells, that could be used as a new model system (vs. animal models) to study human disease. For example, Harder et al has shown through single cell transcriptional profiling of podocytes from kidney organoids, that organoid podocyte lineage cells have two distinct transcriptional states (59). These podocyte transcriptional states are comparable to developing human kidneys: the genes that defined the two different human kidney organoid podocyte clusters were the same genes that defined the two different developing human kidney podocyte clusters. These two distinct transcriptional states of organoid podocytes may represent two developmental stages of podocyte cells, supporting the hypothesis that transcriptional plasticity may be used as a compensatory mechanism in glomerular disease. To further understand the effect of two developmental podocyte stages on glomerular disease, an early glomerular epithelial (EGE) gene signature and mature podocyte gene signature were created from the clusters. Interestingly, many genes in the EGE signature resembled genes dysregulated in diseased glomeruli. Specifically, three genes *LYPD1, PRSS23*, and *CDH6* were differentially regulated in EGE cells and strongly associated with increased proteinuria and loss of kidney function from prior analyses of glomerular tissue in two human kidney disease cohorts (59). These data suggest that podocyte loss or dedifferentiation to an earlier cell type, as suggested by increased EGE gene expression, is associated with CKD, making these newly identified genes involved in glomerular pathology potential targets for investigation.

Organoids have also been used in combination with single cell transcriptomics to better understand specific pathology, such as polycystic kidney disease. Cyst size and total kidney volume in polycystic kidney disease are associated with disease progression and mortality. However, phenotypes in polycystic kidney disease differ by family and mutation, which is an area of continued research. To interrogate different phenotypes of polycystic kidney disease, Czerniecki et al. edited genes of kidney organoids to produce cysts. DropSeq analysis of these gene-edited models showed that inhibition of non-muscle myosin II increased cyst formation, and this process is potentially regulated though polycystin-1, a pathway for future investigation (52).

These examples show that analysis of cell specific transcriptional changes in cell development, health, and disease, can lead to identification of novel genes and pathways that may be relevant to human disease.

## Conclusions and Future Directions

Bulk and single cell transcriptomic profiling techniques each have unique contributions to advancing our understanding of kidney disease. These techniques can help us identify cell types and mechanisms relevant to disease processes that thus far have largely been defined by histopathology, which does not well-capture the underlying biologic heterogeneity of CKD and nephrotic syndromes. As demonstrated in the examples provided in this mini-review, interrogations of the kidney transcriptome have helped researchers understand mechanisms and biomarkers of disease. Exciting applications now and in the near future include further interrogation of these discoveries in animal models, targeting these disease pathways with medications with the goal of delaying progression of CKD, and helping researchers identify subgroups of patients who might be most likely to respond to specific pharmacologic therapies. These approaches may benefit all patients with kidney disease, but in particular pediatric patients with a higher likelihood of a germline genomic mutations have much to potentially gain. Translation of this technology to clinical practice in nephrology is not yet available, however application of similar techniques in this bench to bedside paradigm has revolutionized the field of oncology. Ultimately, the goal of applying transcriptomic analyses to blood, urine, and kidney biopsy specimens of individual patients to better provide targeted treatments for their nephrotic syndrome and CKD is part of the future of nephrology.

## Author Contributions

All authors listed have made a substantial, direct and intellectual contribution to the work, and approved it for publication.

### Conflict of Interest Statement

The authors declare that the research was conducted in the absence of any commercial or financial relationships that could be construed as a potential conflict of interest.
